# A conserved abundant cytoplasmic long noncoding RNA modulates repression by Pumilio proteins in human cells

**DOI:** 10.1038/ncomms12209

**Published:** 2016-07-13

**Authors:** Ailone Tichon, Noa Gil, Yoav Lubelsky, Tal Havkin Solomon, Doron Lemze, Shalev Itzkovitz, Noam Stern-Ginossar, Igor Ulitsky

**Affiliations:** 1 Department of Biological Regulation, Weizmann Institute of Science, Rehovot 76100, Israel; 2 Department of Molecular Genetics, Weizmann Institute of Science, Rehovot 76100, Israel; 3 Department of Molecular Cell Biology,Weizmann Institute of Science, Rehovot 76100, Israel

## Abstract

Thousands of long noncoding RNA (lncRNA) genes are encoded in the human genome, and hundreds of them are evolutionarily conserved, but their functions and modes of action remain largely obscure. Particularly enigmatic lncRNAs are those that are exported to the cytoplasm, including NORAD—an abundant and highly conserved cytoplasmic lncRNA. Here we show that most of the sequence of NORAD is comprised of repetitive units that together contain at least 17 functional binding sites for the two mammalian Pumilio homologues. Through binding to PUM1 and PUM2, NORAD modulates the mRNA levels of their targets, which are enriched for genes involved in chromosome segregation during cell division. Our results suggest that some cytoplasmic lncRNAs function by modulating the activities of RNA-binding proteins, an activity which positions them at key junctions of cellular signalling pathways.

## Abstract (web summary)

The human genome contains thousands of long noncoding RNAs which have been preserved by evolution, through their functions are poorly described. Here the authors show that NORAD binds the Pumilo homologues PUM1 and PUM2 to regulate mRNA levels of genes involved in chromosome segregation.

## Graphical abstract

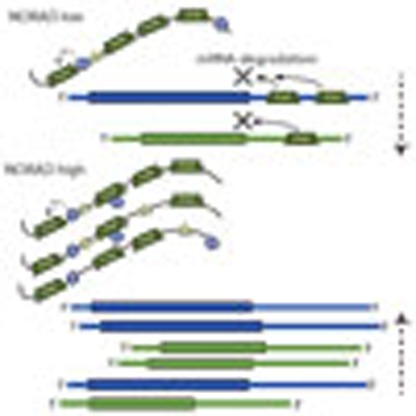

Genomic studies conducted over the past 15 years have uncovered the intriguing complexity of the transcriptome and the existence of tens of thousands of long noncoding RNA (lncRNA) genes in the human genome, which are processed similarly to mRNAs but appear not to give rise to functional proteins^1^. While some lncRNA genes overlap other genes and may be related to their biology, many do not, and these are referred to as long intervening noncoding RNAs, or lincRNAs. An increasing number of lncRNAs are implicated in a variety of cellular functions, and many are differentially expressed or otherwise altered in various instances of human disease^2^; therefore, there is an increasing need to decipher their modes of action. Mechanistically, most lncRNAs remain poorly characterized, and the few well-studied examples consist of lncRNAs that act in the nucleus to regulate the activity of loci found in *cis* to their sites of transcription^3^. These include the XIST lncRNA, a key component of the X-inactivation pathway, and lncRNAs that are instrumental for imprinting processes, such as AIRN^4^. However, a major portion of lncRNAs are exported to the cytoplasm: indeed, some estimates based on sequencing of RNA from various cellular compartments suggest that most well-expressed lncRNAs are in fact predominantly cytoplasmic^1^.

The functional importance and modes of action of cytoplasmic lncRNAs remain particularly poorly understood. Some lncRNAs that are transcribed from regions overlapping the start codons of protein-coding genes in the antisense orientation can bind to and modulate the translation of those overlapping mRNAs^5^, and others have been proposed to pair with target genes through shared transposable elements found in opposing orientations^6^. Two lncRNAs that are spliced into circular forms were shown to act in the cytoplasm by binding Argonaute proteins (in one case, through ∼70 binding sites for a miR-7 microRNA^7^) and act as sponges that modulate microRNA-mediated repression^7^,^8^. Such examples are probably rare, as few circRNAs and few lncRNAs contain multiple canonical microRNA-binding sites^9^. It is not clear whether other cytoplasmic lncRNAs can act as decoys for additional RNA-binding proteins through a similar mechanism of offering abundant binding sites for the factors.

The Pumilio family consists of highly conserved proteins that serve as regulators of expression and translation of mRNAs that contain the Pumilio recognition element (PRE) in their 3′-untranslated regions (3′-UTRs)^10^. Pumilio proteins are members of the PUF family of proteins that is conserved from yeast to animals and plants, and whose members repress gene expression either by recruiting 3′ deadenylation factors and antagonizing translation induction by the poly(A) binding protein^11^, or by destabilizing the 5′ cap-binding complex. The *Drosophila* Pumilio protein is essential for proper embryogenesis, establishment of the posterior-anterior gradient in the early embryo, and stem cell maintenance^12^. Related roles were observed in other invertebrates^10^, and additional potential functions were reported in neuronal cells^13^. There are two Pumilio proteins in humans, PUM1 and PUM2 (ref. 10), which exhibit 91% similarity in their RNA-binding domains, and which were reported to regulate a highly overlapping but not identical set of targets in HeLa cells^14^. Mammalian Pumilio proteins have been suggested to be functionally important in neuronal activity^15^, ERK signalling^16^, germ cell development^17^ and stress response^15^. Therefore, modulation of Pumilio regulation is expected to have a significant impact on a variety of crucial biological processes.

Here, we characterize NORAD—an abundant lncRNA with highly expressed sequence homologues found throughout placental mammals. We show that NORAD is bound by both PUM1 and PUM2 through at least 17 functional binding sites. By perturbing NORAD levels in osteosarcoma U2OS cells, we show that NORAD modulates the mRNA abundance of Pumilio targets, in particular those involved in mitotic progression. Further, using a luciferase reporter system we show that this modulation depends on the canonical Pumilio binding sites.

## Results

### NORAD is a cytoplasmic lncRNA conserved in mammals

**Figure 1:**
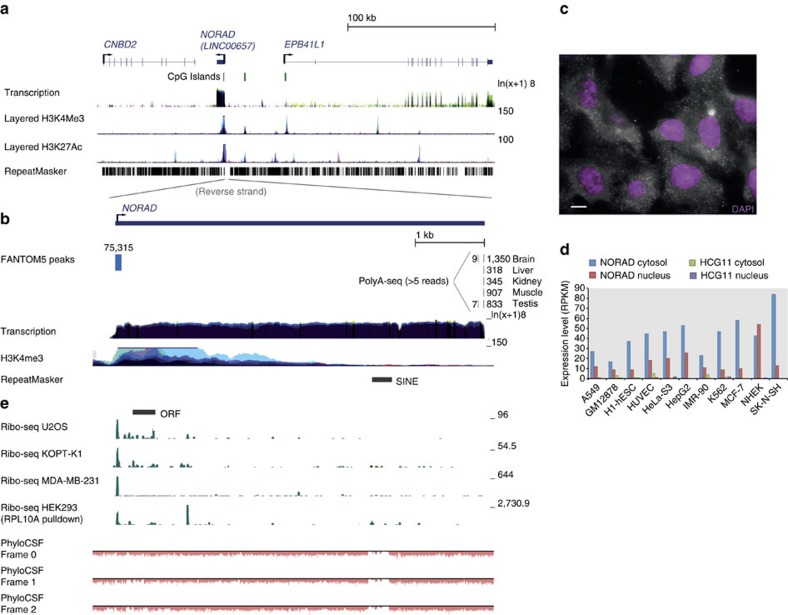
Overview of the human NORAD locus. (**a**) Genomic neighbourhood of NORAD. CpG island annotations and genomic data from the ENCODE project taken from the UCSC genome browser. (**b**) Support for the NORAD transcription unit. Transcription start site information taken from the FANTOM5 project^45^. Polyadenylation sites taken from PolyA-seq data set^46^. ENCODE data sets and repeat annotations from the UCSC browser. (**c**) Predominantly cytoplasmic localization of NORAD by smFISH. Scale bar, 10 μm. See Supplementary Fig. 2 for RNA-FISH following NORAD knockdown. (**d**) Expression levels of NORAD and HCG11 in the ENCODE cell lines (taken from the EMBL-EBI Expression Atlas (https://www.ebi.ac.uk/gxa/home)). (**e**) Support for the noncoding nature of NORAD. Ribosome-protected fragments from various human cell lines (MDA-MB-231 (ref. 24), HEK-293 (ref. 25), U2OS^26^ and KOPT-K1 (ref. 27)) mapped to the NORAD locus as well as PhyloCSF^47^ scores. All PhyloCSF scores in the locus are negative.

In our studies of mammalian lncRNA conservation, we identified a conserved and abundant lincRNA currently annotated as *LINC00657* in human and *2900097C17Rik* in mouse, and recently denoted as ‘noncoding RNA activated by DNA damage' or *NORAD*^18^. *NORAD* produces a 5.3 kb transcript that does not overlap other genes (Fig. 1a), starts from a single strong promoter overlapping a CpG island, terminates with a single major canonical poly(A) site, but is unspliced, unlike most long RNAs (Fig. 1b). Similar transcripts with substantial sequence homology can be seen in EST and RNA-seq data from mouse, rat, rabbit, dog, cow, and elephant. *NORAD* does not appear to be present in opossum, where a syntenic region can be unambiguously identified based on both flanking genes with no evidence of a transcribed gene in between them, and no homologues could be found in more basal vertebrates. NORAD is ubiquitously expressed across tissues and cell lines in human, mouse and dog, with comparable levels across most embryonic and adult tissues (Supplementary Fig. 1) with the exception of neuronal tissues, where NORAD is more highly expressed. In the presently most comprehensive data set of gene expression in normal human tissues, compiled by the GTEX project (http://www.gtexportal.org/), the 10 tissues with the highest NORAD expression all correspond to different regions of the brain (highest level in the frontal cortex with a reads per kilobase per million reads (RPKM) score of 142), with levels in other tissues varying between an RPKM of 78 (pituitary) to 27 (pancreas). Comparable levels were also observed across ENCODE cell lines, with the highest expression in the neuroblastoma SK-N-SH cells (Fig. 1d). The high expression levels of NORAD in the germ cells have probably contributed to the large number of closely related NORAD pseudogenes found throughout mammalian genomes. There are four pseudogenes in human that share >90% homology with NORAD over >4 kb, but they do not appear to be expressed, with the notable exception of HCG11, which is annotated as a lincRNA and is expressed in a variety of tissues but at levels ∼20-times lower than NORAD (based on GTEX and ENCODE data, Fig. 1d). Because of this difference in expression levels, we assume that while most of the experimental methods we used are not able to distinguish between NORAD and HCG11, the described effects likely stem from the NORAD locus and not from HCG11. Using single-molecule *in situ* hybridization (smFISH)^19^ in U2OS cells, we found that NORAD localizes almost exclusively to the cytoplasm (Fig. 1c and Supplementary Fig. 2) and similar cytoplasmic enrichment is observed in other cells lines (Fig. 1d). The number of NORAD copies expressed in a cell is ∼80 based on the RPKM data (assuming an RPKM of 1 roughly corresponds to a single copy per cell) and 68±8 based on the smFISH experiments that we have performed on U2OS cells, with 94% of NORAD copies located in the cytoplasm and 6% in the nucleus.

### NORAD is a bona fide noncoding RNA

NORAD is computationally predicted to be a noncoding RNA by the PhyloCSF (Fig. 1e) and Pfam/HMMER pipelines^20^, with CPAT^21^ and CPC^22^ giving it borderline scores due to the presence of an open reading frame (ORF) with >100aa (see below) and similarity to hypothetical proteins (encoded by NORAD homologues) in other primates. Therefore, we also examined whether NORAD contains any translated ORFs using Ribo-seq data^23^. When examining ribosome footprinting data sets from diverse human cell lines (MDA-MB-231 (ref. 24), HEK-293 (ref. 25), U2OS^26^, and KOPT-K1 (ref. 27)), we did not observe any substantial footprints over any of the ORFs in NORAD, including a poorly conserved 108 aa ORF found close to the 5′-end of the human transcript (Fig. 1e). Interestingly, substantial pileups of ribosome-protected fragments were observed at the very 5′-end of NORAD in all Ribo-seq data sets we examined (Fig. 1e and Supplementary Fig. 3), but those did not overlap any ORFs with either the canonical AUG start codon or any of the common alternative start codons (Supplementary Fig. 3), nor did they encode any conserved amino acid stretches in any of the frames. We conclude that it is highly unlikely that NORAD is translated into a functional protein under regular growth conditions in those cell types, and the footprints observed in Ribo-seq data result from either a ribosome stalled at the very beginning of a transcript, or from a contaminant footprint of a different ribonucleoprotein complex, as such footprints are occasionally present in Ribo-seq experiments^25^,^28^. It remains possible that NORAD is translated in other conditions and contexts.

### NORAD contains at least 12 structured repeated units

**Figure 2:**
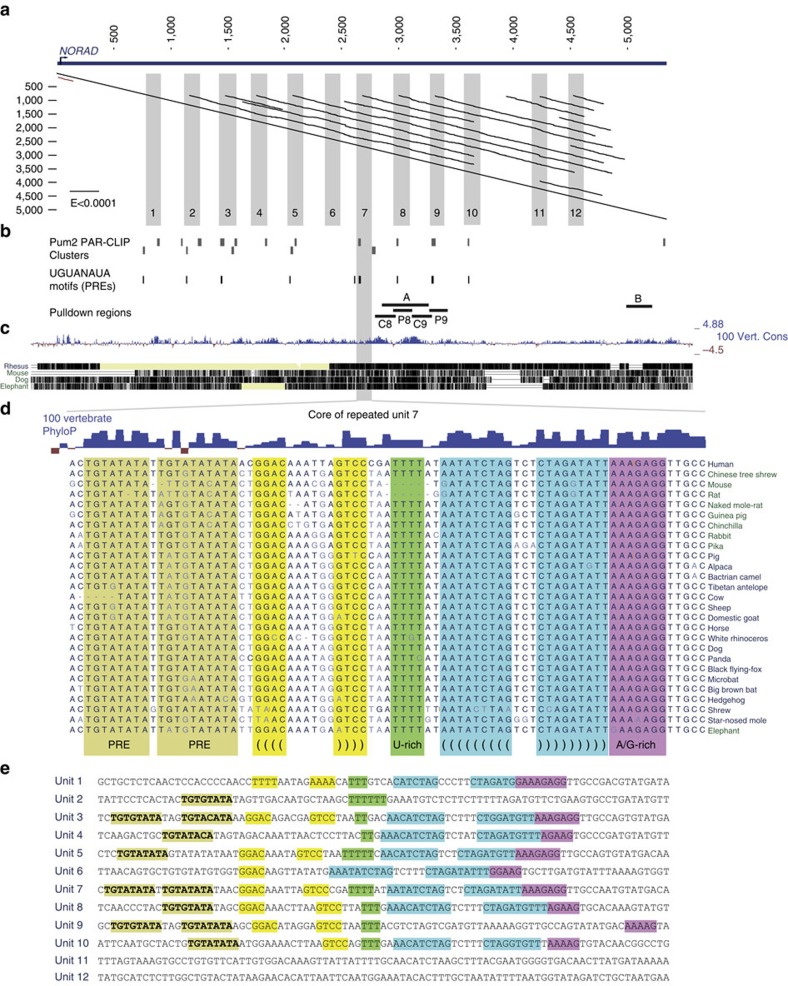
The repeated nature of the NORAD RNA. (**a**) A dotplot computed using plalign^48^ (http://fasta.bioch.virginia.edu/) comparing NORAD with itself. The off-diagonal lines indicate high scoring local alignments between different parts of the sequence. Grey boxes indicate the core of the 12 manually annotated repeated units. (**b**) Clusters identified by PARalyzer^49^ within the NORAD sequence using the PUM2 PAR-CLIP data^30^, positions of PRE UGURUAUA motifs, and regions used for *in vitro* transcription and pulldown of NORAD fragments. (**c**) Sequence conservation of the NORAD locus, with PhyloP^50^ scores for single-base level conservation. (**d**) Detailed conservation of the seventh repeated unit. Shaded regions indicate the five motifs present in most repeated units. (**e**) Core sequences of the human repeated units, with the same shading as in **d**.

When comparing the NORAD sequence to itself, we noticed a remarkable similarity among some parts of its sequence (Fig. 2a). Manual comparison of the sequences revealed that the central ∼3.5 kb of NORAD in human, mouse, and other mammalian species can be decomposed into 12 repeating units of ∼300 nt each. Interestingly, these units appear to have resulted from a tandem sequence duplication that occurred at least 100 million years ago, before the split of the eutherian mammals, as when performing pairwise comparisons, units from different species were more similar to each other than to other units from the same species. Overall, the sequences have diverged to a level where there are no sequence stretches that are strictly identical among all the repeats in human. At the core of the most conserved regions within the repeats we identify four sequence and structure motifs (Fig. 2d,e), some combination of which appears in each of the repeats 1–10: (i) one or two PREs, defined by the consensus UGURUAUA); (ii) a short predicted stem-loop structure with four paired bases and a variable loop sequence. The importance of the structure is supported by the preferential A→G and G→A mutations in the second stem-loop that would preserve the stem (Fig. 2d and Supplementary Fig. 4, also detected by EvoFold^29^); (iii) a U-rich stretch of 2–5 bases; and (iv) a stem-loop structure with eight or nine predicted base pairs. Further sequence conservation is found upstream and downstream of these motifs. Interestingly, the sequences of some of the repeated units, namely 3–5 and 7–9, appear to be more constrained during mammalian evolution than others (Fig. 2c), and those units also tend to contain most of the repeat motifs, with more intact sequences and structures (Fig. 2e).

### NORAD contains multiple functional Pumilio binding sites

**Figure 3:**
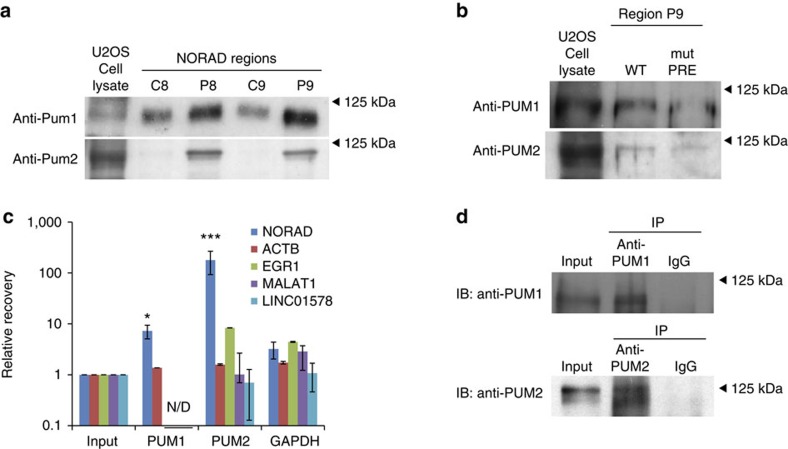
Pumilio proteins bind NORAD. (**a**) Western blots for PUM1 and PUM2 following pulldowns using the indicated *in vitro* transcribed regions (marked in Fig. 2b). (**b**) Western blots for PUM1 and PUM2 following pulldowns using *in vitro* transcribed RNAs from synthetic oligos with WT or mutated PRE. (**c**) Recovery of the indicated transcripts in the input and in the indicated IPs. All enrichments are normalized to GAPDH mRNA and to the input sample as described in Methods. *P<0.05, ***P<0.001 (Tukey's HSD test). Error bars represent s.e.m. based on at least three independent replicates. (**d**) Western blots of the indicated factors in the input and IP samples. HSD, honest significant difference; WT, wild type.

To identify potential protein binding partners of the repeating units and of other NORAD fragments, we first amplified the eighth repeat unit and a region from the 3′-end of NORAD (regions A and B marked in Fig. 2b), transcribed them *in vitro* in the sense (regions A and B) and antisense (region A) orientations using the T7 polymerase with biotinylated UTP bases, incubated the labelled RNA with U2OS cell lysate, and subjected the resulting pulldown material to mass spectrometry. Among the proteins identified as binding different regions of NORAD (Supplementary Data 1) we focus here on two that have predicted binding sites within the repeat units—PUM1 and PUM2, the two verterbrate Pumilio proteins^10^. PUM1 and PUM2 proteins were enriched when we performed similar pulldowns followed by western blots using the PRE-containing regions within repeats 8 and 9 (P8 and P9 in Fig. 2b, Fig. 3a and Supplementary Fig. 5) relative to the adjacent sequences (C8 and C9 marked in Figs 2b and 3a and Supplementary Fig. 5). In addition, enrichment was strongly reduced when one of the two PREs in region P9 had been mutated (Fig. 3b and Supplementary Fig. 5). To gain additional support for a direct interaction between PUM2 and NORAD, we reanalyzed PAR-CLIP data from HEK-293 cells^30^ and found that PUM2 binds at least 17 sites on NORAD (Fig. 2b). These experimentally verified sites (all exhibiting T→C mutations characteristic of PAR-CLIP) overlapped 10 out of the 11 PREs within repeated units 2–10. It is notable that NORAD has an unusual density of PREs encoded in its sequence—there are 17 non-overlapping instances of the UGURUAUA motifs in NORAD compared with 0.38 expected by chance (*P*<0.001, see Methods). The number and density of Pumilio motifs within NORAD are higher than those found in all but one human gene (*PLCXD1*, which has 18 PREs mostly located in transposable elements, compared with 0.12 expected).

To test whether NORAD also co-precipitates with PUM1 and PUM2 in U2OS cells, we performed RNA immunoprecipitation (RIP) of both proteins followed by quantitative real-time PCR, and found a striking enrichment of the NORAD transcript, with a stronger enrichment observed for PUM2 (Methods and Fig. 3c,d). We conclude that NORAD contains at least 17 confident binding sites for Pumilio proteins, most of which appear in conserved positions within the repeated units. Surprisingly, despite the presence of a large number of binding sites, NORAD was not more susceptible to change following PUM1 and PUM2 overexpression or knockdown in U2OS cells (see below) than targets with few PREs, suggesting that NORAD is resistant to substantial degradation by the Pumilio proteins under the tested conditions.

With ∼70 NORAD transcripts per cell (Fig. 1c) and at least 17 functional PREs (Fig. 2b), NORAD possesses the capacity to simultaneously bind ∼1,200 PUM proteins. Quantitative western blot analysis comparing U2OS cell lysates to recombinant proteins expressed in bacteria revealed that PUM1 and PUM2 are expressed at ∼200 and ∼550 copies per cell, respectively (Supplementary Fig. 6). The sites offered by NORAD for Pumilio protein binding, as well as the potential interactions made possible between Pumilio proteins and other NORAD-interacting factors when bound simultaneously, can be sufficient for eliciting a significant effect on the number of functional Pumilio proteins that are available to act as repressors of their other targets.

### NORAD perturbations preferentially affect Pumilio targets

**Figure 4:**
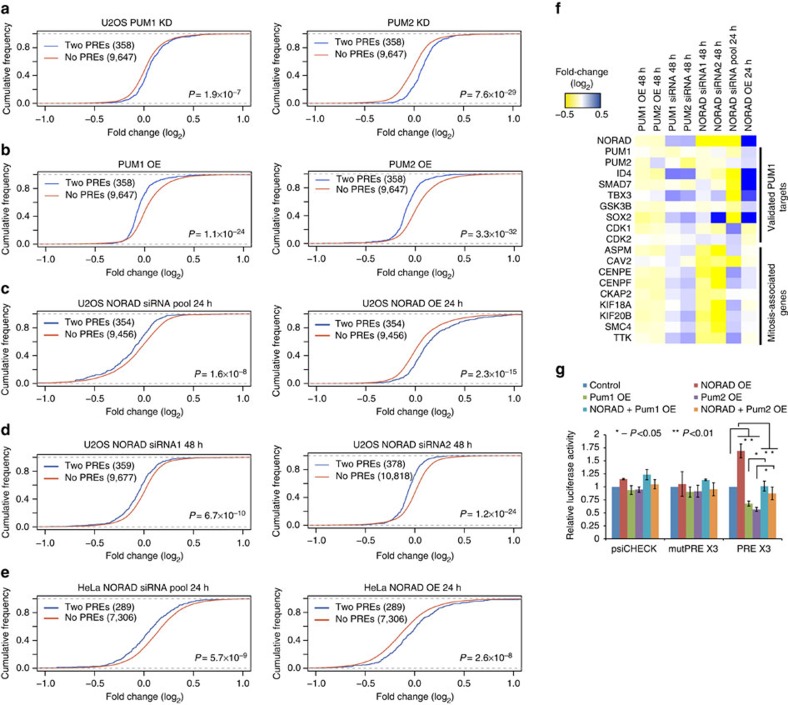
NORAD modulates expression of Pumilio targets. (**a**–**e**) Changes in expression of Pumilio targets compared with controls, following the indicated treatment. Numbers indicate the number of genes in each group that were sufficiently expressed (see Methods). ‘2 PREs' are genes that contain at least two canonical PREs over what is expected by chance in their 3′-UTRs, ‘controls' are those genes that do not contain more sites in their 3′-UTRs than expected by chance. (**f**) Changes in expression of NORAD, PUM1/2, validated targets of PUM1 (ref. 33) and genes with annotated roles in the M phase of the cell cycle and/or the mitotic spindle following the indicated perturbations. (**g**) Changes in the luciferase activity measured from the indicated vectors (top, psiCHECK is a control vector) and RNA expression measured using RT-PCR (bottom) following overexpression of the indicated genes and combinations. Error bars represent s.e.m. based on at least three independent replicates.

As PUM1 and PUM2 are reported to affect mRNA stability^11^,^31^, we next tested whether changes in NORAD expression affect the levels of Pumilio targets. We defined Pumilio target genes as those having at least two extra UGUANAUA sites in their 3′-UTRs over the number of sites expected given the 3′-UTR length of the transcripts. To validate that such genes indeed represent Pumilio targets, we knocked down (KD, Supplementary Fig. 7) and overexpressed (OE) PUM1 and PUM2 separately in U2OS cells and observed significant upregulation and downregulation of predicted Pumilio targets following KD and OE, respectively (Fig. 4a,b). NORAD was then perturbed using either one of two individual siRNAs (siRNA 1 and siRNA 2, Supplementary Fig. 8A) or a pool of four siRNAs (Dharmacon), with the pool yielding ∼4-fold knockdown and individual siRNAs yielding ∼2-fold knockdown (Supplementary Fig. 8A). We obtained consistent effects with the two siRNAs 48 hs after transfection (Supplementary Fig. 8B, Supplementary Data 2), with 51 genes consistently downregulated by at least 20% and 23 genes consistently upregulated by at least 20% after treatment with both siRNAs. The stronger knockdown using a pool of siRNAs (Supplementary Fig. 8A) resulted in more substantial changes in gene expression—584 genes were consistently downregulated by at least 30% in two replicates, and 68 genes were consistently upregulated (Supplementary Data 2). To test the consequences of increased NORAD levels, we cloned NORAD into an expression vector, where its transcription was driven by a CMV promoter, and transfected this vector into U2OS and HeLa cells, which resulted in 2–16-fold NORAD upregulation. Changes following NORAD downregulation at 24 h were strongly inversely correlated with the changes observed 24 h after NORAD OE (Supplementary Fig. 8C and Supplementary Data 2, Spearman *r*=−0.54, *P*<10^−10^), suggesting that the differential expression was indeed driven by changes in NORAD abundance. Strikingly, Pumilio targets were repressed more than controls when NORAD was downregulated, and their expression levels increased more than controls when NORAD was upregulated in both U2OS and HeLa cells (Fig. 4c–e). These differences remained significant after controlling for the increased lengths of the 3′-UTRs of genes bearing Pumilio motifs (Supplementary Fig. 9A) and when considering genes with PUM2 PAR-CLIP clusters in their 3′-UTR as determined in HEK-293 cells (these effects were strongest 48 h after transfection, Supplementary Fig. 9B). Genes with multiple PREs were generally more affected than those with fewer sites (Supplementary Fig. 10A). Differences between Pumilio targets and controls were observed when considering exon-mapping and not when considering intron-mapping reads, pointing at post-transcriptional regulation^32^ (Supplementary Fig. 10B). Lastly, we observed consistent effects in validated PUM1 targets^33^ expressed in U2OS cells (Fig. 4f). These results suggest that hundreds of genes regulated by the two Pumilio proteins are sensitive to NORAD levels, with increased NORAD amounts alleviating repression of Pumilio targets and decreased NORAD amounts increasing repression.

When we inspected the Gene Ontology annotations enriched in the different sets of genes responsive to NORAD perturbations, after correction for multiple testing using TANGO^34^, the only significantly enriched group were genes bound by PUM2 in the PAR-CLIP data and downregulated 48 h after NORAD knockdown. These genes were enriched with categories associated with cell cycle and mitosis, including ‘M phase of the cell cycle' (eight genes; *P*=6.4 × 10^−6^) and ‘Spindle' (eight genes; *P*=1.2 × 10^−7^). Interestingly, these genes were not substantially affected at 24 h after NORAD knockdown or overexpression (Fig. 4f), and enrichments of NORAD targets were also significant when compared with all PUM2-bound targets, suggesting a cumulative, and perhaps cell cycle-dependent, effect of NORAD perturbation on Pumilio targeting of genes involved in mitosis. These results are consistent with the chromosomal instability and mitotic defects observed in other cell types following TALEN-mediated deletion of NORAD^18^.

As Pumilio proteins may affect translation in addition to their effects on mRNA stability, we evaluated the translational consequences of NORAD perturbation after 48 h using Ribo-seq^35^. Consistent with the RNA-seq data, the number of translating ribosomes on Pumilio targets was reduced following NORAD KD (Supplementary Fig. 11A). However, when normalizing for changes in mRNA levels, translation efficiency of Pumilio targets did not appear to be preferentially affected (Supplementary Fig. 11B), suggesting that the main effects of NORAD on Pumilio targets are through effects on mRNA stability rather than translation. This observation is consistent with reports that the mechanism of action of Pumilio proteins is through interaction with deadenylation complexes^11^,^31^ that can first affect protein translation, but eventually results in mRNA decay.

### NORAD regulation is dependent on the canonical PREs

To test whether regulation of Pumilio targets depends on the presence of canonical PREs, we utilized a luciferase reporter vector containing three strong PREs as well as a control reporter with mutated sites, in which the three 5′-UGUACAUA-3′ motifs were mutated to 5′-ACAACATA-3′ (mutPRE)^11^,^31^. As expected, overexpression of PUM1 or PUM2 proteins in U2OS cells led to reduced luciferase activity in a PRE-dependent manner (Fig. 4g). Overexpression of NORAD, on the other hand, alleviated the repression of the PRE-containing luciferase mRNA, without affecting mutPRE-containing mRNA. Simultaneous OE of NORAD and the Pumilio proteins abrogated both effects, an observation consistent with our model that the effect of NORAD on Pumilio targets is mediated through Pumilio proteins (Fig. 4g and Supplementary Fig. 12A). Knockdowns of NORAD or PUM1 or PUM2 failed to yield a consistent effect on luciferase activity (Supplementary Fig. 12B), possibly because of the limited knockdown efficiency using siRNAs (Supplementary Fig. 12C) or through feedback regulation of PUM1 or PUM2 on their own mRNA. Overall, these results indicate that the NORAD-dependent changes in abundance of Pumilio targets are likely mediated through canonical PREs.

## Discussion

To our knowledge, NORAD comprises the first example of a lncRNA that contains multiple highly conserved consensus binding sites for an RNA-binding protein (RBP), and that is required for proper regulation of the RBP targets at physiological levels. One particularly interesting question that remains open is the functional importance and roles of the other conserved elements found in the NORAD repeats, and in particular the two predicted hairpin structures, as such conserved secondary structures are rarely detectable in lncRNAs^1^. It is possible that these structural elements serve as binding sites for other RBPs, whose binding may either facilitate the binding of PUM1 and PUM2 to NORAD or affect PUM1 or PUM2 protein stability or activity. We note that while the overall number of binding sites offered by NORAD for PUM1 and PUM2 (∼1,200) is comparable to the Pumilio abundance in U2OS cells, which we estimate at ∼200 and ∼550 copies per cell for each of PUM1 and PUM2, respectively (Supplementary Fig. 6), these sites are outnumbered by the sites present in other expressed mRNAs, and therefore it is possible that NORAD does not merely titrate Pumilio proteins away from their other targets but rather induces a change in their activity, potentially by serving as a scaffold for interaction of Pumilio proteins with other factors. Potentially interesting candidates for interacting with NORAD repeats that were identified in the mass spectrometry analysis are known RBPs, such as IGF2BP1/2/3, XRN2 and PABPN1. In addition, we observed that the interferon response pathway proteins IFIT1/2/3/5 and their downstream companion PKR could bind NORAD sequence. IFIT proteins were observed to bind the antisense of the NORAD eighth repeat unit, suggesting that they may recognize a structural element rather than a primary sequence within the repeat, whereas PUM1 and PUM2 bound only the sense sequence, consistent with their known sequence specificity. We were so far unable to substantiate interactions with IFIT1 and PKR by reciprocal pulldown experiments.

While this manuscript was under review, Mendell and colleagues described a role for NORAD and PUM2 in ensuring chromosomal segregation fidelity in various human cells^18^. Further studies will be required in order to uncover the full spectrum of physiological consequences of the regulation of Pumilio targets by NORAD, but the enrichment of cytokinesis-related genes among the Pumilio targets that are sensitive to NORAD levels suggests that NORAD may modulate regulation of chromosomal segregation during mitosis by Pumilio, and might even affect the conserved roles of Pumilio in regulating asymmetric cell divisions during embryonic development. An intriguing question is whether the relatively high levels of NORAD in U2OS cells correspond to a basal state, in which NORAD exerts a minimal effect on PUM1 and PUM2 that is increased when stimuli increase NORAD expression, or to a state where NORAD actively buffers substantial regulation by PUM1 and PUM2. Most results point to the former scenario, as relatively modest overexpression of NORAD resulted in stronger effects on Pumilio activity than its knockdown. Another possibility suggested by the enrichment of cell cycle regulated genes among the most prominent NORAD and Pumilio targets is that this regulation is cell cycle-dependent.

## Methods

### Cell culture

Human cell lines U2OS (osteosarcoma, obtained from the ATCC) and HeLa (cervical carcinoma, obtained from the ATCC) were routinely cultured in DMEM containing 10% fetal bovine serum and 100 U penicillin/0.1 mg ml^−1^ streptomycin at 37 °C in a humidified incubator with 5% CO_2_.

### Plasmids and siRNAs

Plasmid transfections were performed using polyethyleneimine (PEI)^36^ (PEI linear, M*r* 25,000 from Polyscience Inc). To overexpress NORAD, the full transcript of the lincRNA was amplified from human genomic DNA (ATCC NCI-BL2126) using the primers 5′-TGCCAGCGCAGAGAACTGCC-3′ (Fw) and 5′-GGCACTCGGGAGTGTCAGGTTC-3′ (Rev), and cloned into a ZeroBlunt TOPO vector (Invitrogen), and then subcloned into the pcDNA3.1(+) vector (Invitrogen). PUM1 and PUM2 were overexpressed using pEF-BOS vectors^37^,^38^ (a kind gift of Prof. Takashi Fujita). As controls in overexpression experiments, we used pBluescript II KS+ (Stratagene). Plasmids were used in the amount of 0.1 μg per 100,000 cells in 24-well plates for 24 h before cells were harvested. The luciferase experiments employed the following plasmids: pGL4.13; psiCheck-1 containing 3X wild-type PRE, which is underlined in the following sequence, 5′-TTGTTGTCGAAAATTGTACATAAGCCAA-3′; psiCheck-1 containing 3X mutated PREs: 5′-TTGTTGTCGAAAATACAACATAAGCCAA-3′ and psiCheck-1 with no PRE, all previously described^11^,^31^ (a kind gift of Dr Aaron Goldstrohm). pGL4.13 was used in the amount of 5 ng per 20,000 cells in 96-well plates, while the different psiCheck-1 plasmids were used in the amount of 15 ng per 20,000 cells in 96-well plates.

Gene knockdown was achieved using siRNAs directed against NORAD, PUM1 and PUM2 genes (all from Dharmacon, Supplementary Table 1), while as control we used the mammalian non-targeting siRNA (Lincode Non-targeting Pool, Dharmacon), at final concentration of 50 nM for 24 or 48 h before further experimental procedures. The transfections into U2OS cells were conducted using PEI.

siRNA transfection into HeLa cells were conducted using 100 nM siRNA and Dharmafect (Dharmacon) transfection reagent and using siRNA buffer only as a control, and transfection of pCDNA3.1-NORAD was into HeLa cells was peformed using Lipofectamine 2,000.

### Real-time PCR analysis of gene expression

Total RNA was isolated using TRI reagent (MRC), followed by reverse transcription using an equal mix of oligo dT and random primers (Quanta), according to the manufacturer's instructions. For determination of all genes levels real-time PCR was conducted using Fast SYBR qPCR mix (Life Technologies). The primer sets used for the different genes are listed in Supplementary Table 2. The assays contained 10–50 ng sample cDNA in a final volume of 10 μl and were run on AB quantitative real-time PCR system ViiA 7 (Applied Biosystems). All genes expression levels in the different treatments are represented relative to their relevant control (ΔCt) and normalized to GAPDH gene levels (ΔΔCt).

### Fluorescent *in situ* hybridization

Probe libraries were designed according to Stellaris guidelines and synthetized by Stellaris as described in Raj *et al.*^19^. Libraries consisted of 48 probes 20 nt each, complementary to the NORAD sequence according to the Stellaris guidelines (Supplementary Table 3). Hybridizations were done overnight at 30 °C with Cy5 labelled probes at a final concentration of 0.1 ng μl^−1^. DAPI dye (Inno-TRAIN Diagnostik Gmbh) for nuclear staining was added during the washes. Images were taken with a Nikon Ti-E inverted fluorescence microscope equipped with a × 100 oil-immersion objective and a Photometrics Pixis 1,024 CCD camera using MetaMorph software (Molecular Devices, Downington, PA). The image-plane pixel dimension was 0.13 μm. Quantification was done on stacks of 4–12 optical sections with Z-spacing of 0.3 μm. Dots were automatically detected using a custom Matlab program, implementing algorithms described in Raj *et al.*^19^. Briefly, the dot stack images were first filtered with a three-dimensional Laplacian of Gaussian filter of size 15 pixels and standard deviation of 1.5 pixels. The number of connected components in binary thresholded images was then recorded for a uniform range of intensity thresholds and the threshold for which the number of components was least sensitive to threshold selection was used for dot detection. Automatic threshold selection was manually verified and corrected for errors. Background dots were detected according to size and by automatically identifying dots that appear in more than one channel (typically<1% of dots) and were removed.

### RNA pulldown

Templates for *in vitro* transcription were generated by amplifying the desired sequences from cDNA or from synthetic oligos, adding the T7 promoter to the 5′-end for sense and 3′-end for the antisense sequence (see Supplementary Table 2 for primer sequences). In addition, protein pulldown was performed using an oligo with the sequence of repeat #9 (5′-GTCTGCATTTTCATTTACTGTGCTGTGTATATAGTGTATATAAGCGGACATAGGAGTCCTAATTTACGTCTAGTCGATGTTAAAAAGGTTGCCAGTATATGACAAAAGTAGAATTAGTAAACTACTACATTGAGTACACTTTGTGTTAAAATTCATAGGGA)-3′ and an oligo that contains a mutation in its PRE (5′-GTCTGCATTTTCATTTACTGTGCT**ACA**TATATAGTGTATATAAGCGGACATAGGAGTCCTAATTTACGTCTAGTCGATGTTAAAAAGGTTGCCAGTATATGACAAAAGTAGAATTAGTAAACTACTACATTGAGTACACTTTGTGTTAAAATTCATAGGGA-3′) using the primers from Supplementary Table 2. Biotinylated transcripts were produced using the MEGAscript T7 *in vitro* transcription reaction kit (Ambion) and Biotin RNA labelling mix (Roche). Template DNA was removed by treatment with DnaseI (Quanta). Cells were lysed in buffer containing 20 mM Tris^·^HCl pH 7.5, 150 mM NACl, 1.5 mM MgCl_2_, 2 mM DTT, 0.5% Na-Deoxycholate, 0.5% NP-40) for 15 min on ice. The extract was cleared by centrifugation at 21,130*g* at 4 °C for 20 min. Extract containing 0.5–2 mg of protein was incubated with 2–20 pmole of biotynylated transcripts. The pulldown products were analysed by mass spectrometry and western blots. For the mass spectometry the formed RNA-protein complexes were precipitated by Streptavidin-sepharose high-performance beads (GE Healthcare). Recovered proteins were then resolved on a 4–12% Express Page gradient gel (GeneScript), visualized by silver staining. The entire lane was extracted and analysed using mass spectrometry analysis as described^37^. Briefly, peptide fragments were separated using a Nanosep3KD micro centrifuge tube (Pall, USA) and MS measurements were performed using a nano-electrospray ionization quadrupole time-of-flight (ESI-Q-TOF) instrument (Applied Biosystems, Foster City, CA). The spectra were searched against human database with the use of the MASCOT (Matrix Science, London, UK).

Alterantively, the recovered proteins were separated on a 10% SDS–polyacrylamide gel electrophoresis gel, and used for western blotting with anti-PUM1 or anti-PUM2 antibodies (Bethyl Laboratories; anti-Pum1: A300-201A; anti-Pum2 A300-202A). In addition, RNA was isolated using TRI reagent from equal portion of the different protein-RNA pulldown complexes. This RNA was analysed using RT-PCR for loading control.

### RNA immunoprecipitation (RIP)

Immunoprecipitation (IP) of endogenous ribonucleoprotein complexes from whole-cell extracts was performed as described by Yoon *et al.*^39^. In brief, cells were incubated with lysis buffer (20 mM Tris^·^HCl at pH 7.5, 150 mM NACl, 1.5 mM MgCl_2_, 2 mM DTT, 0.5% Na-deoxycholate, 0.5% NP-40, complete protease inhibitor cocktail (Sigma) and 100unit per ml RNase inhibitor (EURx)) for 15 min on ice and centrifuged at 15,870*g* for 15 min at 4 °C. Part of the supernatants was saved as total cell lysate input. The rest, containing 1–2 mg protein extract, was incubated for 2–3 h at 4 °C in gentle rotation with protein A/G magnetic beads (GeneScript). The beads were pre-washed and coated with antibodies against GAPDH (SantaCruz SC-32233 Biotechnology, diluted 1:1,000), PUM1 and PUM2 (Bethyl,Laboratoris, A300-201A and A3000202A respectively, diluted 1:1,000) at 4 °C in gentle rotation overnight. As a negative control, we incubated the magnetic beads-antibodies complexes with lysis buffer. The beads were washed five times with lysis buffer, each time separated by magnetic force. The remaining mixture of magnetic beads-antibodies-protein-RNA complexes were separated as half were mixed with sample buffer and boiled at 95C for 5 min for further analysis by Western blot and the other half was incubated with 1 mg ml^−1^ Proteinase K for 30 min at 37C with gentle shaking to remove proteins. The remaining RNA was extracted by TRI reagent. The RNAs isolated from the IP materials were further assessed by RT-qPCR analysis as follows: 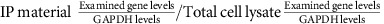. Western blot was used in order to verify that the desired protein was indeed precipitated.

### Ribosome profiling

U2OS cells, transfected with siRNAs, were lysed in lysis buffer (20 mM Tris^·^HCl pH 7.5, 150 mM KCl, 5 mM MgCl2, 1 mM dithiothreitol, 8% glycerol) supplemented with 0.5% triton, 30 U ml^−1^ Turbo DNase (Ambion), and 100 μg ml^−1^ cycloheximide, and ribosome-protected fragments were then generated, cloned and sequenced as previously^23^. Briefly, the lysate was cleared by centrifugation, treated with RNase I for 45 min and then loaded on a sucrose cushion. RNA was extracted cushion pellet using TRI reagent and small RNA fragments (28–34 bp) were size selected via gel purification. These RNA fragments were then dephosphorylated, ligated with an adaptor to their 3′-end, and reverse transcribed. The resulting cDNA was circularized and PCR amplified introducing Illumina sequencing adaptors.

### RNA-seq and data analysis

Strand-specific mRNA-seq libraries were prepared from U2OS cells using the TruSeq Stranded mRNA Library Prep Kit (Illumina), according to the manufacturer's protocol, and sequenced on a NextSeq 500 machine to obtain at least 23 million 75 nt reads. Strand-specific mRNA-seq libraries for HeLa cells were prepared as described^40^. Briefly, the RNA was fragmented with base hydrolysis and fragments between 26 and 32 nt were gel-extracted. Adaptors containing fixed sequences were ligated to the 3′- and 5′-ends of the RNA fragments followed by additional gel extractions, and after cDNA synthesis, Illumina sequencing adaptors were added using PCR. Reads were aligned to the human genome (hg19 assembly) using STAR Aligner^41^, and read counts for individual genes (defined as overlapping sets of RefSeq transcripts annotated with their Entrez Gene identifier) were counted using htseq-count^42^ and normalized to reads per million aligned reads. For counting intron-mapping reads, htseq-count was used to count reads mapping to the whole-gene locus, and the exon-mapping reads were then subtracted for each gene. Only genes with an average RPM of at least 50 normalized reads across the experimental conditions were considered, and fold changes were computed after addition of a pseudo-count of 0.1 to the RPM in each condition. The raw read counts and the computed fold-changes appear in Supplementary Data 2.

### Sequence analyses

Whole-genome alignments were obtained from the UCSC genome browser. Expected numbers of PREs were computed by applying dinucleotide-preserving permutations to the sequences and counting motif occurrences in the shuffled sequences. 3′-UTR-length-matched control targets were selected by dividing the genes into 10 bins based on 3′-UTR lengths and randomly sampling the same numbers of genes not enriched with Pumilio target sites as the number of genes enriched with sites from each bin.

### Luciferase assays

Reporter gene activity was measured as previously described^43^. Briefly, 20,000 cells were plated in a 96-well plate. After 24 h cells were co-transfected with pGL4.13 as an internal control and with the indicated psiCheck plasmids. In addition, the cells were transfected with the various siRNAs or plasmids (as described above). Luciferase activity was recorded 48 h post transfection using the Dual-Glo Luciferase Assay System (Promega) in the Micro plate Luminometer device (Veritas). A relative response ratio, from RnLuc signal/FFLuc signal, was calculated for each sample. Percent of change is relative to the control siRNA or control plasmid.

### Determination of copy number of PUM1 and PUM2 in U2OS cells

PUM1 and PUM2 were expressed in bacteria. Briefly, PUM1 and PUM2 cDNA were cloned into a modified version of pMal-C2 expression vector (a kind gift from the laboratory of Prof. Deborah Fass) by restriction free cloning resulting in a MBP-6His-PUM constructs. The plasmids were transformed into Rosetta-R3 bacteria (Novagen). Bacteria were grown in 15 ml 2YT media in the presence of 100 μg ml^−1^ ampicillin and 50 μg ml^−1^ chloramphenicol to OD600≈0.6. Recombinant protein expression was induced for 18 h at 16 °C by 500 μM IPTG.

Bacterial pellet was resuspended in 5 ml of lysis buffer B (100 mM NaH_2_PO_4_, 10 mM Tris, 8 M Urea, pH8) and incubated on a rotating shaker for 90 min at room temperature. The extract was cleared by centrifugation (10,000*g*, 20 °C for 30 min). Cleared extract was incubated for 60 min with 1 ml of 50% Nickel beads slurry (Ni-NTA His^·^Bind Rasin, Novagen) and the extract bead mix was loaded onto an empty column. The column was washed twice with wash buffer C (100 mM NaH_2_PO_4_, 10 mM Tris, 8 M Urea, pH6.3) and the bound proteins were eluted four times in 500 μl of elution buffer D (100 mM NaH_2_PO_4_, 10 mM Tris, 8 M Urea, pH5.9), followed by four times in 500 μl of elution buffer E (100 mM NaH_2_PO_4_, 10 mM Tris, 8 M Urea, pH4.5). Sample of each fraction was run on SDS–polyacrylamide gel electrophoresis and analysed by Coomassie blue staining. To determine the quantity of PUM1 and PUM2 copies per cell we calibrated a standard curve using the purified bacterial expressed PUM proteins and then plotted the protein expression levels from a lysate extracted from a measured number of cells.

### Statistics

All results are represented as an average ±s.e.m. of at least three independent experiments. Statistics was performed as Student's *t*-test, Wilcoxon rank-sum test or analysis of variance with Tuckey's *post hoc* test for three or more groups to be compared. In all results *P<0.05, **P<0.01, ***P<0.001. Plots were prepared using custom R scripts. Gene Ontology enrichment analysis was performed using the WebGestalt server^44^ and corrected for multiple testing using TANGO^34^, using all the expressed genes as background set and Benjamini–Hochberg correction for multiple testing.

### Data availability

All data presented in this work is available from the authors upon request. All sequencing data has been deposited to the GEO database (Accession GSE79804).

## Additional information

**How to cite this article:** Tichon, A. *et al.* A conserved abundant cytoplasmic long noncoding RNA modulates repression by Pumilio proteins in human cells. *Nat. Commun.* 7:12209 doi: 10.1038/ncomms12209 (2016).

## Supplementary Material

Supplementary Information Supplementary Figures 1-12 and Supplementary Tables 1-3

Supplementary Data 1 Proteins identified as binding to different regions of NORAD using mass spectrometry

Supplementary Data 2 Differentially expressed genes following various perturbations.
