# Supplementary material for: A conserved abundant cytoplasmic long noncoding RNA modulates repression by Pumilio proteins in human cells
Source: Nat Commun. 2016 Jul 13;7:12209. doi: 10.1038/ncomms12209 (PMC4947167; doi:10.1038/ncomms12209)
Supplement: Supplementary Information — Supplementary Figures 1-12 and Supplementary Tables 1-3 [file ncomms12209-s1.pdf]

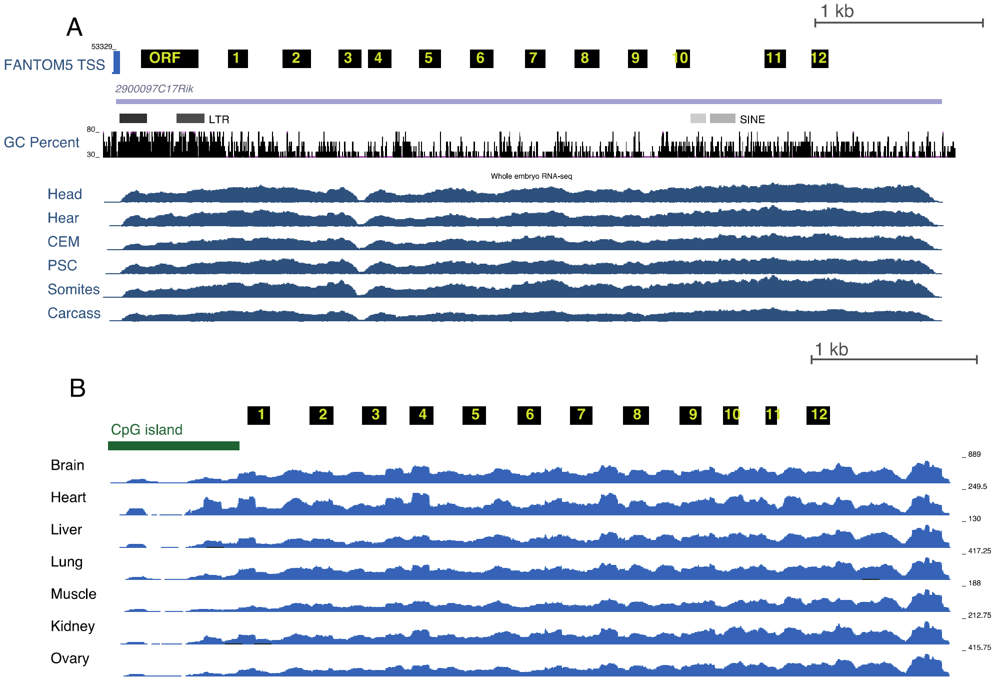

Supplementary Figure 1. NORAD expression in mouse (A) and dog (B). The black boxes indicate the position of the regions alignable to the 12 repeat units in the human genome. Annotated transposable elements in mouse are from the UCSC genome browser (no repeats are annotated in the corresponding region in the dog genome). Mouse transcription evidence (non-strand-specific) is taken from ref[43]. Dog transcription evidence (strand-specific) taken from SRA accession SRP009687.

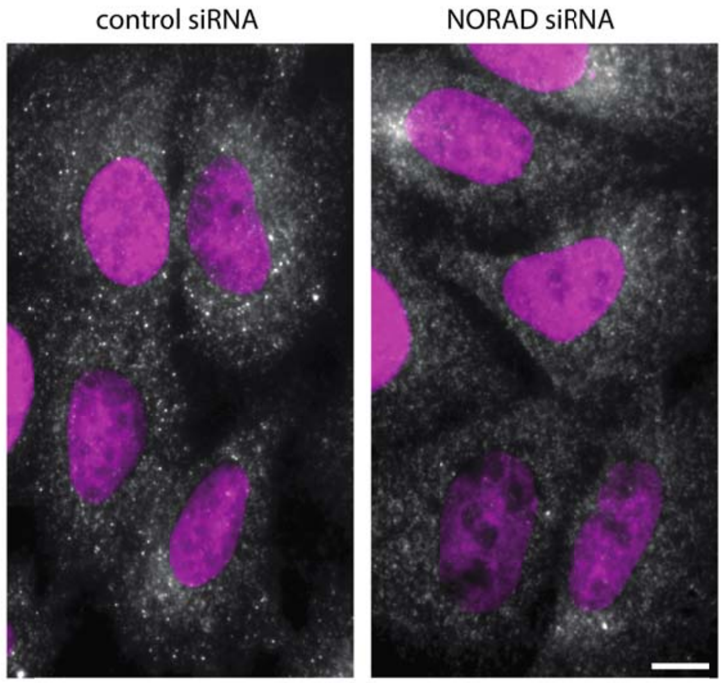

	Average number of NORAD transcripts per cell	standard error
control siRNA	83.395	10.016
NORAD siRNA	16.462	1.384

Supplementary Figure 2. Specificity of FISH probes for NORAD. Single-molecule RNA-FISH of NORAD in U2OS cells transfected with control siRNA and siRNA pool against NORAD. Scale bar is 5µm. Difference between control and NORAD siRNA is significant by Wilcoxon rank sum test with $p<0.01$.

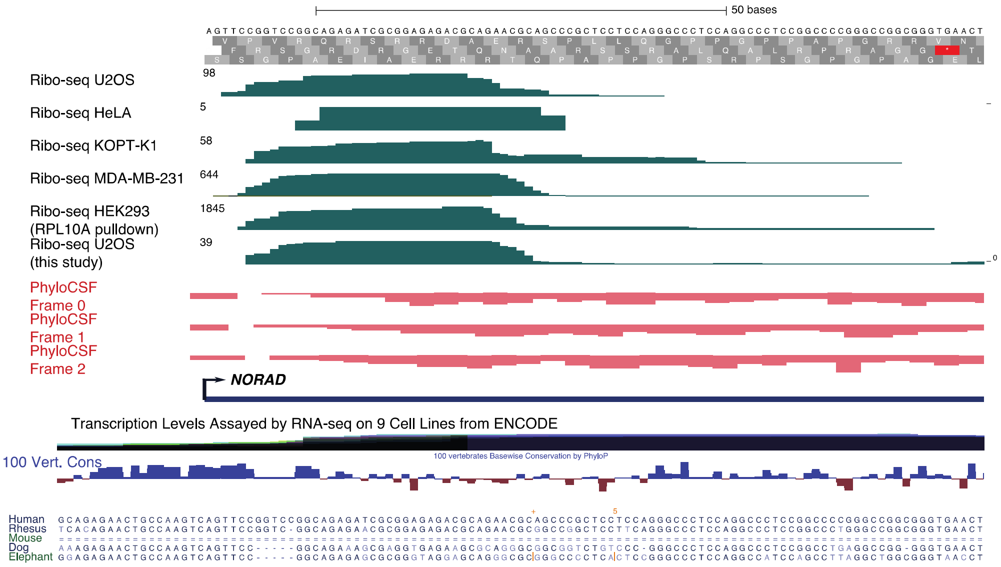

Supplementary Figure 3. The 5' end of NORAD. The top panel shows the sequence of the 5' end of NORAD and its potential translation in the three possible frames. Ribosome footprinting reads mapping to this region from various cell types are shown with the number of alignable reads in each dataset. All PhyloCSF scores in this region are negative.

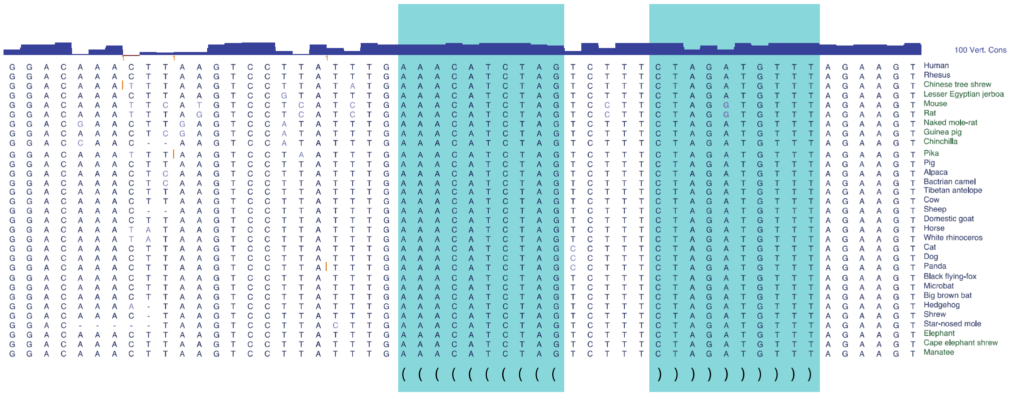

Supplementary Figure 4. Conservation of the longer hairpin motif in repeat unit 8.

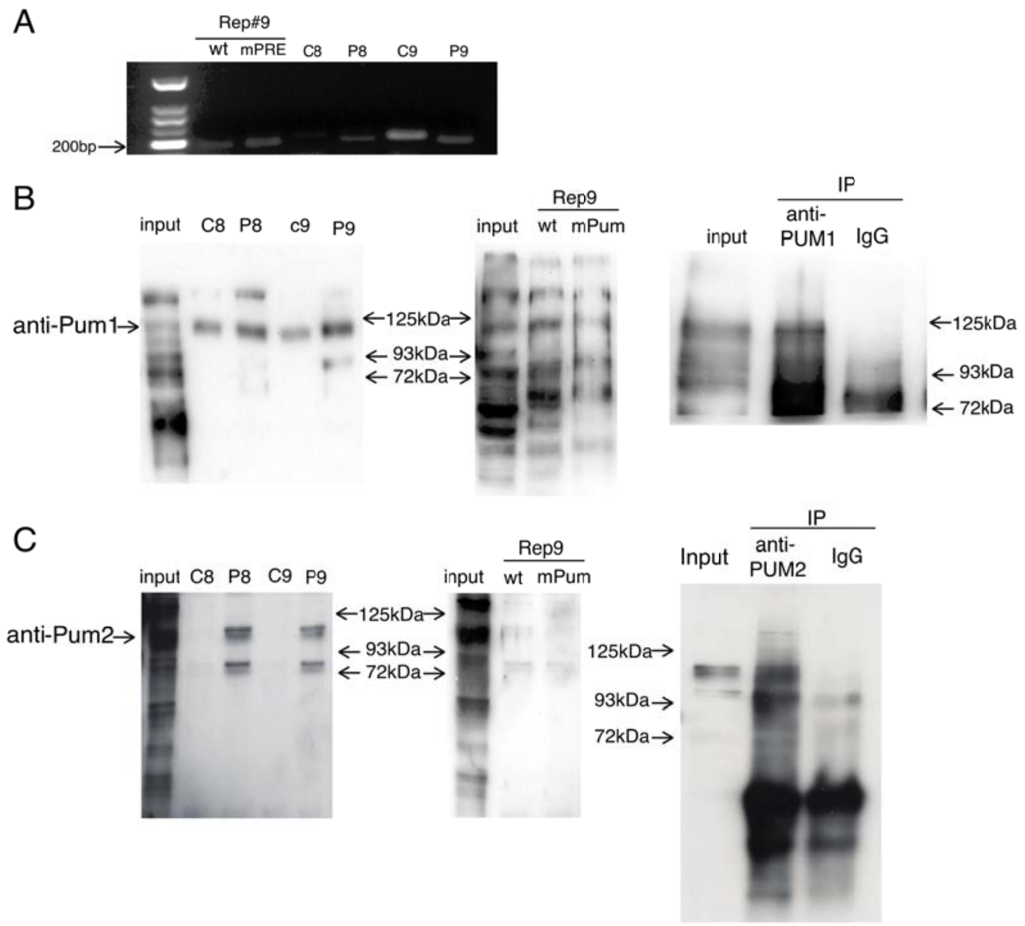

Supplementary Figure 5. Controls and full blots for experiments shown in Figure 3. (A) RNA loading control for Figure 3A-B. RT-PCT products amplified from the RNA substrates used for pulldown. **(B-C)** Full Western blots for PUM1 (B) and PUM2 (C) for experiments in Figure 3.

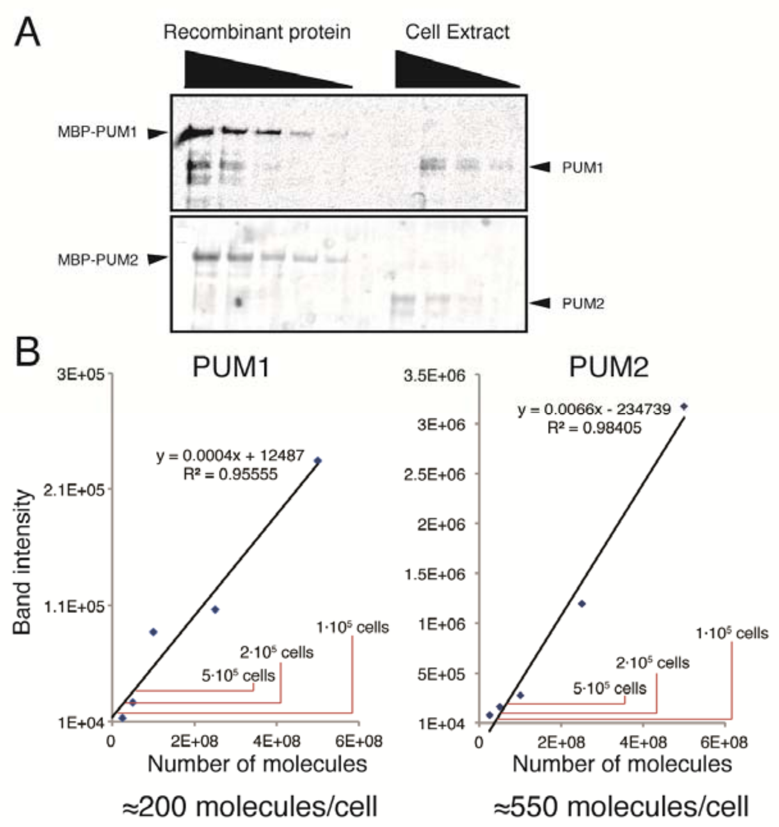

Supplementary Figure 6. Estimation of the copy numbers of PUM1 and PUM2 in U2OS cells. See Methods for experimental details.
A: Decreasing amounts of recombinant PUM1 (top) and PUM2 (bottom) as well as whole cell extract from U2OS cells were analyzed by western blotting. Bands were detected using the Azure c600 imaging system and quantified using the Azure spot software (Azure Biosystems). **B:** Quantification of blot shown in A. Blue rectangles represent the intensities of the recombinant proteins, red line indicates the intensities of bands of Pumillio from extracts made from the indicated cell number.

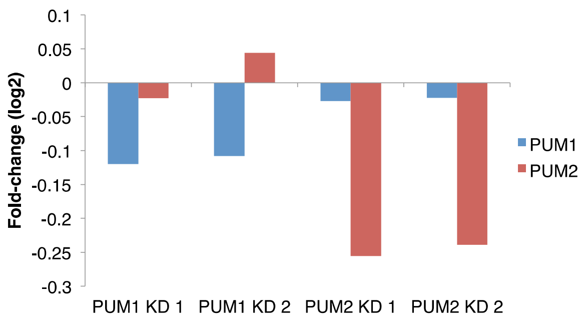

Supplementary Figure 7. Reduction in mRNA levels of PUM1 and PUM2 following siRNA transfections. Fold changes computed from RNA-seq data following each knockdown (KD).

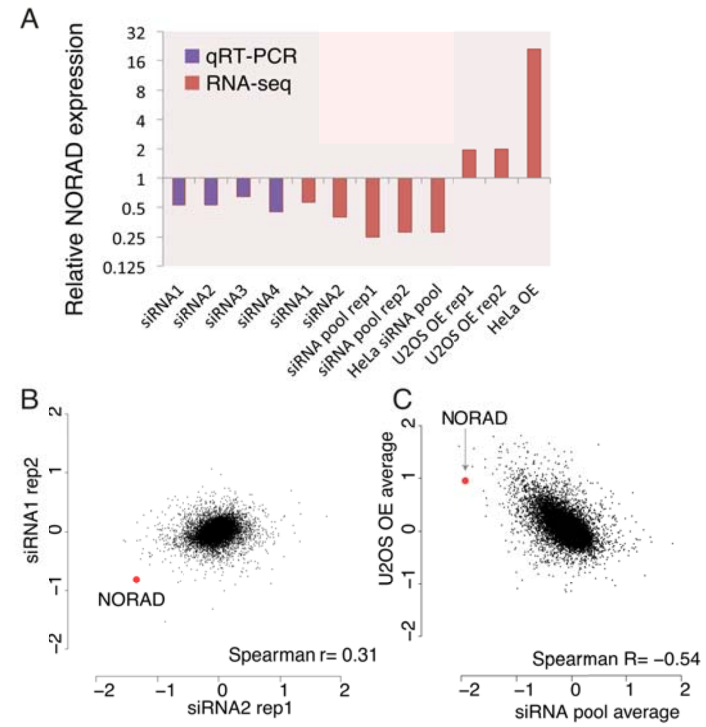

Supplementary Figure 8. (A) Changes in NORAD expression following knockdown using siRNAs or overexpression (OE) from a plasmid, as measured by either qRT-PCR or RNA-seq. **(B)** Correlation of fold changes observed 48 hr after transfection of two independent siRNAs against NORAD. **(C)** Correlation of fold changes observed 24 hr after transfection of a pool of four siRNAs against NORAD or a NORAD over-expression vector.

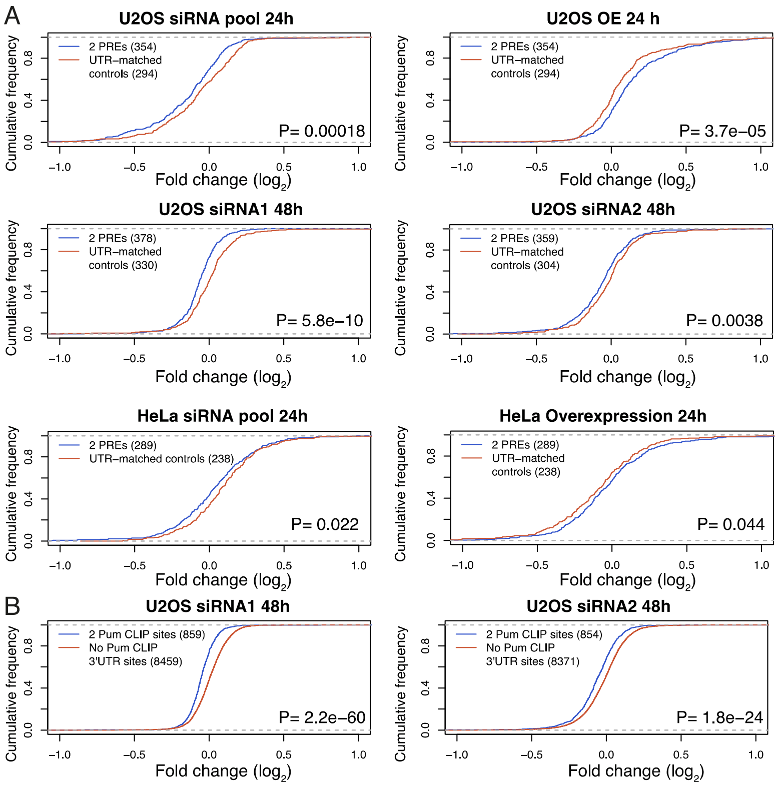

Supplementary Figure 9. (A) Fold changes in gene expression following in the indicated treatments for Pumilio targets and control genes with similar 3' UTR lengths, but no enrichment for PREs. **(B)** Fold changes in gene expression of genes containing at least two PAR-CLIP clusters in HEK93 Pum2 PAR-CLIP experiment and control genes possessing no clusters in that dataset.

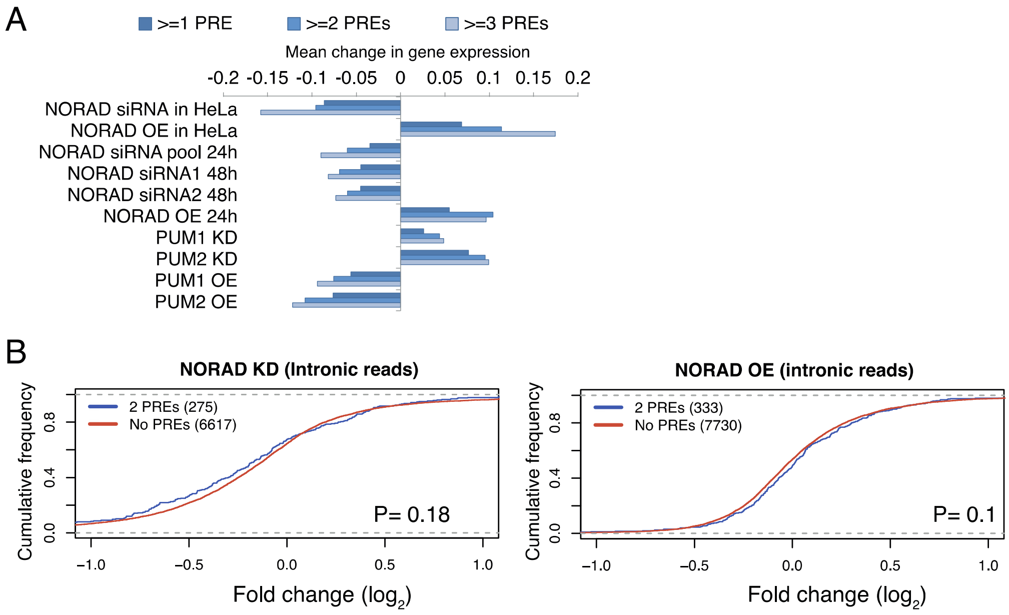

Supplementary Figure 10. (A) Genes with multiple Pumilio binding sites are more strongly regulated by NORAD and Pumilio proteins. Plotted is the mean change in gene expression of genes with the indicated number of Pumilio binding sites in their 3'UTRs (averaged across all the transcripts of a gene). **(B) Changes in gene expression following NORAD perturbations are post-transcriptional.** Changes in gene expression measured using only intron-mapping reads in Pumilio targets and control genes (defined as in Figure 4).

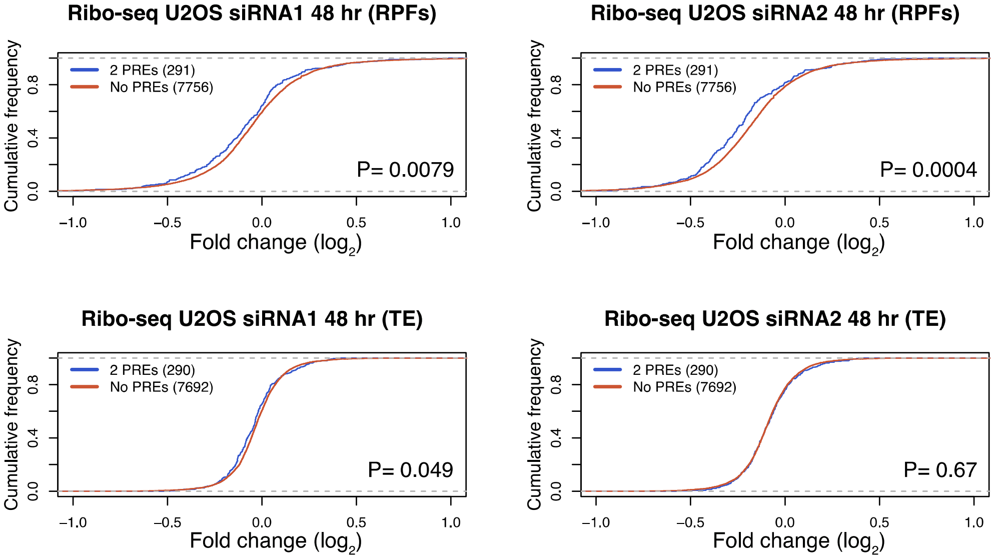

Supplementary Figure 11. Fold changes in numbers of ribosome-protected fragments (RPF) (top) or translational efficiencies (TE) (ribosome protected fragments normalized by RNA-seq reads, bottom) 48 hr after transfection of two independent siRNAs for genes with and without enrichment for PREs.

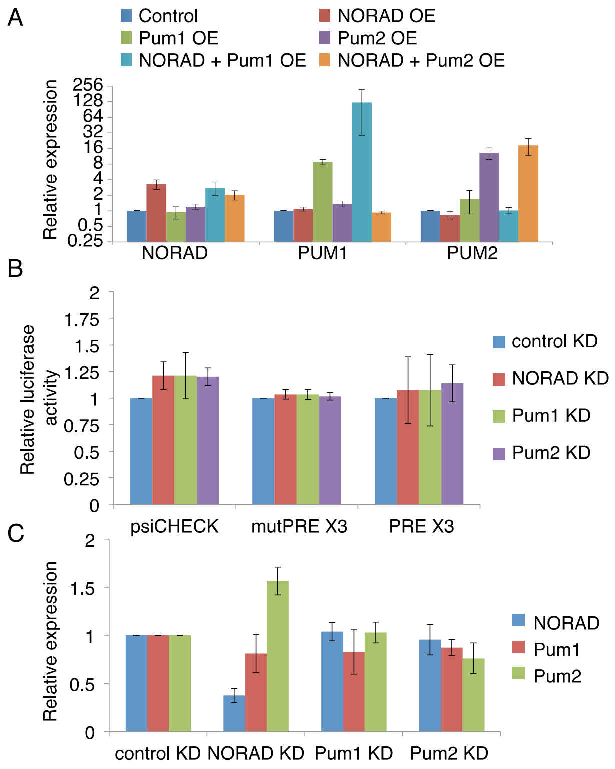

Supplementary Figure 12. Effect of over-expression and knockdowns of NORAD, PUM1, and PUM2 on RNA levels and luciferase reporter activity. (A) qRT-PCR measurements of the indicated genes following transfection of the indicated plasmids. Each experiment was normalized to GAPDH expression and to the control plasmid transfection. (B) Luminescence measured when transfecting the indicated plasmids together with the indicated perturbations. Each experiment was normalized to co-transfected Firefly luciferase and to the control transfection (control siRNA). (C) qRT-PCR measurements of the indicated genes following the indicated perturbations. Each experiment was normalized to GAPDH expression and to the control transfection (control siRNA).

Supplementary Table 1: siRNA sequences. siRNAs #1-4,9-16 where used in U2OS cells, and siRNAs #5-8 in HeLa cells.

ID	*Gene*	*Sequence*	*Note*
siRNA #1	NORAD	CUGUGUAUAUAGCGGACAA	Lincode SMARTpool
siRNA #2	NORAD	CAUCUAAGCUUUACGAAUG	Lincode SMARTpool
siRNA #3	NORAD	AGUGCACAAUGUAGGUUAA	Lincode SMARTpool
siRNA #4	NORAD	CGACCCAAGCCUCGACGAA	Lincode SMARTpool
siRNA #5	NORAD	ACGUCUAGUCGAUGUUAAA	ON-TARGETplus SMARTpool siRNA J-038095-16
siRNA #6	NORAD	AGUGCACAAUGUAGGUUAA	ON-TARGETplus SMARTpool siRNA J-038095-15
siRNA #7	NORAD	AUUCAAUGCUAGUGUGUAU	ON-TARGETplus SMARTpool siRNA J-038095-14
siRNA #8	NORAD	GGAAAGAGGUUGCCGACGU	ON-TARGETplus SMARTpool siRNA J-038095-13
siRNA #9	Pum1	GGUCAGAGUUUCCAUGUGA	ON-TARGETplus SMARTpool siRNA J-014179-05
siRNA #10	Pum1	GGAGGAGGCGGCUAUAAUA	ON-TARGETplus SMARTpool siRNA J-014179-06
siRNA #11	Pum1	GGAGAUAAGCUAGGAGAUU	ON-TARGETplus SMARTpool siRNA J-014179-07
siRNA #12	Pum1	CGGAAGAUCGUCAUGCAUA	ON-TARGETplus SMARTpool siRNA J-014179-08
siRNA #13	Pum2	CUGAAGUAGUUGAGCGCUU	ON-TARGETplus SMARTpool siRNA J-014013-17
siRNA #14	Pum2	GCAGAGUAAUUCAGCGCAU	ON-TARGETplus SMARTpool siRNA J-014013-18
siRNA #15	Pum2	GACAAAUGGUAGUGGUCGA	ON-TARGETplus SMARTpool siRNA J-014013-19
siRNA #16	Pum2	AGACAUAACAGUAACACGA	ON-TARGETplus SMARTpool siRNA J-014013-20

Supplementary Table 2: Primer sequences

Gene	*Fw primer*	*Rv primer*
NORAD	5'- AGCGAAGTCCCGAACGACGA	5'- TGGGCATTTCCAACGGGCCAA
PUM1	5'-AAAAACCTGAGAAGTTTGAATTGT	5'- GCAAGACCAAAAGCAGAGTTG
PUM2	5'-CGCTGACCTCACTGGCCCA	TGGAGCAACCACTTGCCCGT
EGR1	5'-GCACCTGACCGCAGAGTCTTT	5'-TGGGGTAACTGGTCTCCACC
MALAT1	5'- ATGCAGTTGTCTTGACTTCAGGTCTGT	5'- ACACCAGCAAAATGTACTCAGCTTCA
LINC01578	5'- GGCTGCAGTGATACATGCGA	5'- ACTCCAACGTCTCAGTCTTCAA
GAPDH	5'- AGAAGGCTGGGGCTCATTTG	5'- GGTGCTAAGCAGTTGGTGGT
ACTB	5'- TTCCTTCCTGGGCATGGAGT	5'- AATGCCAGGGTACATGG GG
**Region A for**	TTCTAATACGACTCACTATAGGGCAC	GACAATGGTCAATGTGCCTCC

**in vitro transcription**	GTGCCTATATCCATCAGGT	
**Region B for in vitro transcription**	TAATACGACTCACTATAGGG TCTAGAGGCGTGTTGCCATT	CTGTGTGTAGGCACAACATCC
**Region P8 for in vitro transcription**	TTCTAATACGACTCACTATAGGGACC ATTGTTACATGTGTGTAGTTT	TCATATCAAAAGGATAGCTACAAAA
**Region P9 for in vitro transcription**	TTCTAATACGACTCACTATAGGGTCT GCATTTTCATTTACTGTGCT	TCCCTATGAATTTTAACACAAAGT
**Region C8 for in vitro transcription**	TTCTAATACGACTCACTATAGGGTTG TAGAAAGGCTGTCTTCTG	CTACACACATGTAACAATGGT
**Region C9 for in vitro transcription**	TTCTAATACGACTCACTATAGGGTGA AATATTGTCTTGGAAATTGA	AGCACAGTAAATGAAAATGCAGA
**P9 and mutP9 oligo amplification**	TTCTAATACGACTCACTATAGGGTCT GCATTTTCATTTACTGTG	TCCCTATGAATTTTAACACAAAGT

Supplementary Table 3: smFISH probe sequences

1	tgggaaagagaggttcgctg
2	tagaatgaagaccaaccgcc
3	cattctaccatttctctctt
4	atacacaggccttccataaa
5	ccatctagaagggctagatg
6	tttatcatacgtcggcaacc
7	gtcatctccagaagacatgt
8	aaacgtggacgtatcgcttc
9	tcaggcacttcagaacatct
10	cccccaaaaatatttcctat
11	aacaggatggcatagagctc
12	gtcaattaggactcgtctgt
13	atacactggcaacctcttta
14	atgtgaacattctggcctag
15	catacatcgggcacttctaa
16	tactgttcacaaaggtggct
17	taccttttgcaatttcttcc
18	atacactggcaacctcttta
19	ctaaatgtggccattttggc
20	caaacagcatttcccatcag
21	catacattggcaacctcttt
22	tttcagaagacagcctttct
23	aacattctggtctagaaccc
24	gacttaagtttgtccgctat
25	gggtactgctcagagaattg
26	ccctgaaccagcacaaatat
27	aattaggactcctatgtccg
28	tgtcatatactggcaacctt
29	cttaggggggttttaacaa
30	acagctataagccatctgta
31	aggaacattctggcctagaa
32	cctctactgttaacctacat
33	acaaatgcttagaggggtgt
34	attccattcccaaatgcaac
35	tgaatagtctgcattcgctc
36	aatctcaggagcacagaacc
37	ccatcgtctagatatggaga
38	tgtagacctgtggttatcat
39	cccattcgtaaagcttagat
40	catgtccaagatgtatccta
41	tgacatacactgctcagagg
42	acacatctgcatacatctct
43	acagccaagagatgcataca
44	aatgctgcattcccttaatg

45	aagcattggcagagttctga
46	aaatggcaacacgcctctag
47	gggactgagactgtacagta
48	attcttctagatcctgtgtg
